# Methylglyoxal Induces Platelet Hyperaggregation and Reduces Thrombus Stability by Activating PKC and Inhibiting PI3K/Akt Pathway

**DOI:** 10.1371/journal.pone.0074401

**Published:** 2013-09-13

**Authors:** Karin Hadas, Voahanginirina Randriamboavonjy, Amro Elgheznawy, Alexander Mann, Ingrid Fleming

**Affiliations:** 1 Institute for Vascular Signalling, Centre for Molecular Medicine, Goethe University, Frankfurt am Main, Germany; 2 Endokrinologikum Frankfurt, Frankfurt am Main, Germany; Texas A & M, Division of Cardiology,, United States of America

## Abstract

Diabetes is characterized by a dysregulation of glucose homeostasis and platelets from patients with diabetes are known to be hyper-reactive and contribute to the accelerated development of vascular diseases. Since many of the deleterious effects of glucose have been attributed to its metabolite methylgyloxal (MG) rather than to hyperglycemia itself, the aim of the present study was to characterize the effects of MG on platelet function. Washed human platelets were pre-incubated for 15 min with MG and platelet aggregation, adhesion on matrix-coated slides and signaling (Western blot) were assessed *ex vivo*. *In vivo*, the effect of MG on thrombus formation was determined using the FeCl_3_-induced carotid artery injury model. MG potentiated thrombin-induced platelet aggregation and dense granule release, but inhibited platelet spreading on fibronectin and collagen. *In vivo*, MG accelerated thrombus formation but decreased thrombus stability. At the molecular level, MG increased intracellular Ca^2+^ and activated classical PKCs at the same time as inhibiting PI3K/Akt and the β3-integrin outside-in signaling. In conclusion, these findings indicate that the enhanced MG concentration measured in diabetic patients can directly contribute to the platelet dysfunction associated with diabetes characterized by hyperaggregability and reduced thrombus stability.

## Introduction

Diabetes mellitus is associated with the accelerated development of cardiovascular diseases which are the primary cause of morbidity and mortality in patients. Indeed, it is well accepted that the pathogenesis and progression of the vascular complications of diabetes are characterized by the development of endothelial dysfunction and an alteration in platelet function. Although a dysregulation of glucose homeostasis is one of the hallmarks of diabetes, the intracellular mechanism linking hyperglycemia and platelet hyperreactivity are not fully understood.

One consequence of elevated plasma glucose concentrations is the increased formation of methylglyoxal (MG) [1]; a highly reactive dicarbonyl metabolite; that is generated endogenously by the nonenzymatic degradation of the glycolytic intermediates, dihydroxyacetone phosphate and glyceraldehyde-3-phosphate [2]. Normally, plasma levels of MG are maintained at a low levels by the glyoxalase I [3,1], however, this pathway is impaired in diabetes [4], which results in the accumulation of MG [5,6]. Most of the deleterious effects of MG have been attributed to the formation of advanced glycation end-products (AGEs) and the subsequent activation of the AGE receptor; RAGE, which then initiates the vascular and neuronal complications of diabetes. However, MG can also exert effects that are independent of the AGE-RAGE pathway. For example, MG was found to depolarize sensory neurons and induce the post-translational modification of the voltage-gated sodium channel Na _v_1.8 thus resulting in an increased electrical excitability of nociceptive neurons [7]. Moreover, MG has been reported to enhance the formation of neutrophil-platelet aggregates [8] and to increase platelet hydrogen peroxide formation [9]. This study set out to determine the effects of MG on platelet function in vitro and in vivo and to determine the molecular pathways targeted by the metabolite.

## Materials and Methods

### Reagents

Type I collagen, fibronectin, and the anti-PKCα and PKC-β antibodies were from BD transduction laboratories (Heidelberg, Germany). Thrombin was from Hemochrom Diagnostica (Essen, Germany), U46619 was from Cayman Chemical (Biomol, Hamburg, Germany). The anti-Ser19-MLC20, anti-total Akt and anti-phospho Ser473- Akt antibodies were from Cell Signaling (New England Biolabs, Frankfurt, Germany), the anti-Tyr747 β3 integrin antibody was from Invitrogen (Karlsruhe, Germany), the anti-β3 integrin antibody from Chemicon (Hofheim, Germany) and Ro-318220 (bisindolylmaleimide IX) was from Alexis (Lörrach, Germany). All other compounds and antibodies were from Sigma-Aldrich (Steinheim, Germany).

#### Preparation of glycated albumin

Glycated-human serum albumin (G-HSA) was prepared by incubating native HSA with 250 mmol/L D-glucose at 37°C for 4 weeks in Ca^2+^-Mg^2+^-free phosphate buffered saline (PBS) containing protease inhibitors and antibiotics. Control-HSA was processed the same way in the absence of glucose. At the end of the incubation period, the solutions were dialyzed against PBS at 4°C for 24 h to remove unincorporated glucose, and/or antibiotics and then sterile filtered.

### Healthy donors and diabetic patients

A total of 19 patients (8 women, 11 men; mean age 41±7.3 years; hemoglobin (Hb) A_1c_ over 7.4%, fasting plasma glucose 8.4±0.8 mmol/L and fasting plasma insulin 18.5 ±10.9 mU/L) with type 2 diabetes mellitus attending the clinic for routine control visits were included in the present study. All patients were treated with insulin alone or in combination with metformin. Nondiabetic, age-matched subjects (17 women, 23 men; mean age 34.9±8.3 years; HbA_1c_ 5.3±0.56%; fasting plasma glucose 5.2±0.2 mmol/L and fasting plasma insulin 8.17±6.4 mU/L) who had not taken any medication known to interfere with platelet aggregation for at least 10 days before the experiment served as the control group.

### Ethics statement

The study protocol was approved by the ethics committee of the Goethe University Hospital (No. E 61/09 geschäfts Nr 86/09) and all of the participants gave written informed consent.

### Animals

C57BL/6 mice (6-8 weeks old) from Charles River (Sulzfeld, Germany) were used for the present study. Mice were housed in conditions that conform to the *Guide for the Care and Use of Laboratory Animals* published by the U.S. National Institutes of Health (NIH publication no. 85-23). Both the University Animal Care Committees and the Federal Authorities for Animal Research, Regierungspräsidium Darmstadt (Hessen, Germany; F28/44) approved the study protocol.

### Platelet isolation

Human platelets were obtained by centrifugation (900*g*, 7 minutes) of platelet-rich plasma, as described [10]. The resulting pellet was washed in Ca^2+^-free HEPES buffer (mmol/L: NaCl, 136; KCl, 2.6; MgCl_2_, 0.93; NaH_2_PO_4_, 3.26; glucose, 5.5; HEPES, 3.7; pH 7.4 at 37°C) and samples were either lysed for Western Blot analysis, or resuspended in HEPES buffer to a density of 4x10^8^ platelets/mL for the measurement of intracellular Ca^2+^ or platelet aggregation as outlined [11].

### Immunoblotting

Washed human platelets in suspension or platelets adherent on fibronectin or collagen were solubilized in Triton X-100 lysis buffer and Triton X-100 soluble proteins were separated by SDS-PAGE and subjected to Western blotting and visualized by enhanced chemiluminescence using a commercially available kit (Amersham, Freiburg, Germany), as described [10].

### Platelet aggregation

Aggregation of washed human platelets or platelet-rich plasma (4x10^8^ platelets/mL) was measured using an 8-channel aggregometer (PAP8, Mölab, Germany).

### Calcium measurements

Changes in [Ca^2+^]_i_ were determined by measuring fura-2 fluorescence. Platelets were loaded with fura-2/AM (5 µmol/L, 1 hour, 37°C) and [Ca^2+^]_i_ was determined fluorometrically by continuous rapid alternating excitation from dual monochromators set at 340 and 380 nm (Deltascan, Photon Technology) as described [11].

### ATP measurement

The release of ATP was determined using a luciferin/luciferase ATP kit (Enliten^®^ ATP assay system; Promega) as described [10].

### Methylglyoxal measurement

Platelet-poor plasma (PRP) from healthy donors and diabetic patients was separated from whole blood and stored at -80°C until analysis. MG concentration was measured using a commercially available ELISA kit (MBS704102 from MyBiosource).

### P-selectin expression

Washed human platelets were incubated with either solvent or MG for 15 minutes and then stimulated with either solvent or the thrombin receptor agonist (TRAP, 10 µM, 5 minutes). After stimulation, platelets were fixed with formalin (2% in PBS v/v, 15 minutes), washed and incubated with FITC-conjugated anti-P-selectin Ab, or control mouse IgG for 15 minutes at room temperature. After washing surface expression of P-selectin was analyzed using a FACSCalibur flow cytometer (BD Biosciences).

### Platelet adhesion and spreading assays

Static adhesion assays were performed as described [12]. Briefly, washed human platelets were incubated with either solvent or MG (1 mmol/L) in the absence or in the presence of different stimuli. Platelet suspensions (5x10^4^ platelets/µL) were seeded on glass slides (µ-Slide 8 well, ibidi, Martinsried, Germany), coated with either fibronectin (100 µg/ml) or collagen (1.8 ng/ml) and incubated at 37°C for 60 minutes. Nonadherent platelets were removed by washing and adherent and spread platelets were fixed. Images were captured by a AxioCam MRm on a Cell Observer microscope (Zeiss, Jena, Germany) and analyzed using the imaging software AxioVision 4.8 (Zeiss, Jena, Germany).

### In vivo thrombosis formation

Mice were anesthetized by intraperitoneal injection of ketamine and xylazine and placed on a heated mat. MG (1 mmol/L) was injected into the jugular vein and the fluorescent dye 3,3’-dihexyloxacarbocyanine iodide (DIOC_6_; Ivitrogen, Darmstadt, Germany) was also administered (5 µL of a 100 µmol/L solution/g body weight) after 25 minutes to allow visualization of the thrombus. Thereafter, a segment of the right carotid artery was exposed and injury was induced by the topical application of FeCl_3_ for 2 minutes (Whatmann paper 1 mm^2^ soaked with 0.2 µL of 10% FeCl_3_) as described [12]. The artery was then rinsed with saline and thrombus formation was monitored for 30 minutes by placing the carotid artery under a fluorescence microscope equipped with a camera (AxioScope, Carl Zeiss, Jena, Germany). Fluorescent images were acquired sequentially (1 image/second) and thrombus size was quantified using AxioVision 4.7 imaging software (Carl Zeiss).

### Statistical analysis

Data are expressed as mean ± SEM and statistical evaluation was performed using Student’s t test for unpaired data or one-way analysis of variance (ANOVA) followed by a Bonferroni t test where appropriate using Prism software (GraphPad). Values of *P*<0.05 were considered statistically significant.

## Results

### Effects of methylglyoxal on platelet aggregation induced by thrombin and collagen

MG was measured in PRP from 10 healthy subjects and 10 diabetic patients using an ELISA kit. We were unable to detect MG in any of the samples studied. However, given that other methods measured millimolar concentration of MG in plasma from patients with type 2 diabetes, we chose to use 1 mmol/L MG for short-time stimulation in order to simulate the postprandial burst of MG encountered in diabetic patients.

**Figure 1:**
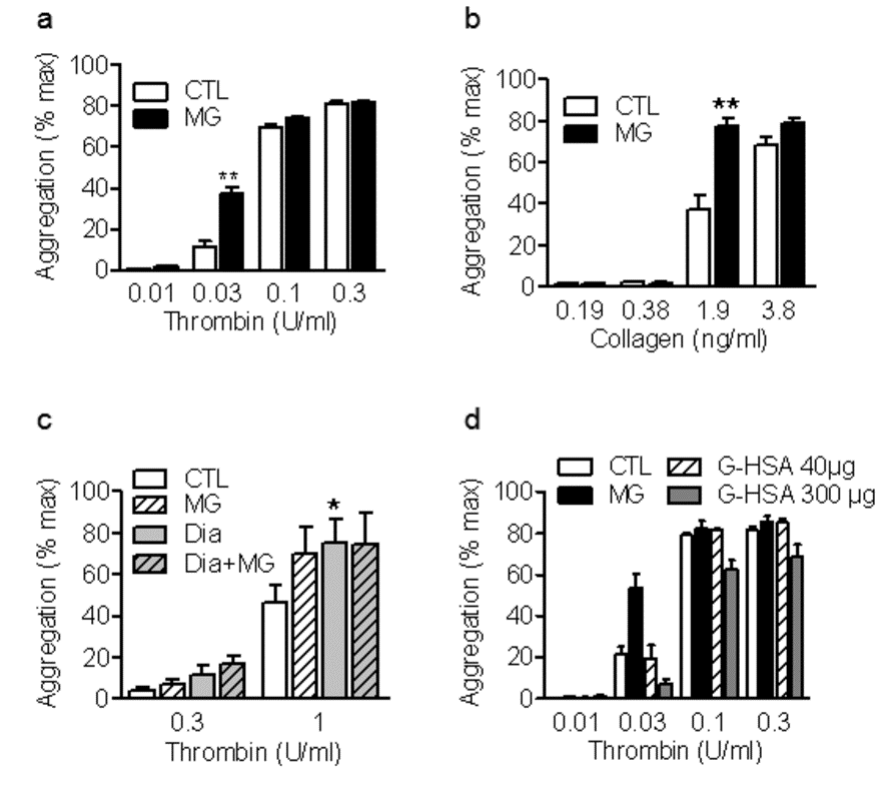
Effect of MG on platelet aggregation. Aggregation of washed human platelets treated with either solvent (CTL) or methylglyoxal (MG, 1 mmol/L, 15 minutes) prior to the stimulation with either (a) thrombin or (b) collagen. (c) Thrombin-induced aggregation of platelet rich plasma from healthy (CTL) or diabetic patients (Dia) in the absence or in the presence of solvent or MG. (d) Aggregation of washed human platelets treated with either solvent (CTL), methylglyoxal (MG, 1 mmol/L, 15 minutes) or glycated human serum albumin (G-HAS, 40 and 300 µg, 15 minutes) prior to the stimulation with thrombin. The graphs summarise the data from at least 6 different individuals; *P<0.05, **P<0.01 versus CTL.

The addition of MG (1 mmol/L) to washed human platelets induced a small but significant aggregation (10.1±0.9% of the maximum response to thrombin; n=8, P<0.01). D-mannitol failed to affect aggregation indicating that the response to MG could not simply be attributed to an osmotic change (Figure S1). When added prior (15 minutes) to other platelet agonists, MG potentiated the aggregation to thrombin and collagen (Figure 1A& B). Interestingly, when the response to MG was assessed in PRP, we found it enhanced the response to thrombin in samples from healthy donors but had no effect on samples from diabetic patients (Figure 1C). In order to determine the consequences of RAGE activation on platelet responses, washed human platelets were treated with glycated human serum albumin (G-HSA). The AGE did not reproduce the effects of MG i.e. did not potentiate the aggregation induced by a low concentration of thrombin. However, high concentrations of G-HSA tended to inhibit thrombin-induced aggregation (Figure 1D). An effect we also observed with the native HSA and may be related to the albumin itself rather than the glycation (data not shown). 

### Effects of MG on Ca^2+^ and Platelet Degranulation

**Figure 2:**
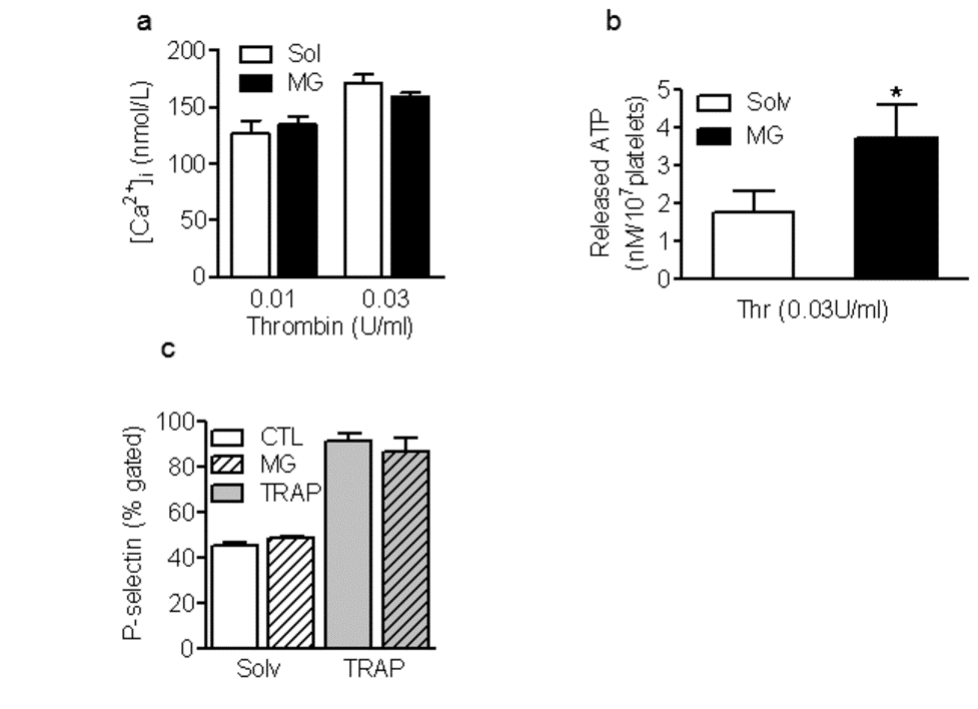
Effect of MG on platelet [Ca^2+^]_i_ and degranulation. (a) Increase in [Ca^2+^]_i_ measured in washed human platelets treated with either solvent (CTL) or methylglyoxal (MG, 1 mmol/L, 15 minutes) prior to the stimulation with thrombin. (b) Effect of MG pre-treatment on the thrombin (0.03U/ml)-induced release of ATP and (c) on the TRAP-induced surface expression of P-selectin. The graphs summarise the data from at least 6 different individuals; *P<0.05, **P<0.01 versus CTL.

Given that the MG-induced increase in aggregation may be related to altered Ca^2+^ signaling we assessed platelet Ca^2+^ responses to thrombin in fura-2-loaded washed human platelets. Consistent with the small effect on aggregation, MG elicited a small (68±4 nmol/L; n=6, P<0.01) but sustained (over 15 minutes) increase in Ca^2+^ over basal levels. However, MG did not significantly alter the Ca^2+^ response to thrombin (Figure 2A). Given that an increase in [Ca^2+^]_i_ is prerequisite for platelet degranulation and thus the potentiation of platelet activation induced by low concentrations of agonists, we assessed the effects of MG on the secretion of dense and α-granules. MG alone did not affect the release of ATP but it did enhance the release of ATP induced by thrombin (Figure 2B). MG also failed to increase the surface expression of P-selectin or affect the responses to the thrombin receptor agonist TRAP (Figure 2C) suggesting that MG did not influence α-granule secretion.

### Methylglyoxal stimulates the membrane translocation of PKCα/β and the phosphorylation of MLC-20

Given the central role played by protein kinase C (PKC) in platelet activation [13] and the fact that PKC activity is enhanced in diabetic platelets [14], we determined whether the effects of MG could be linked to a change in PKC activity.

**Figure 3:**
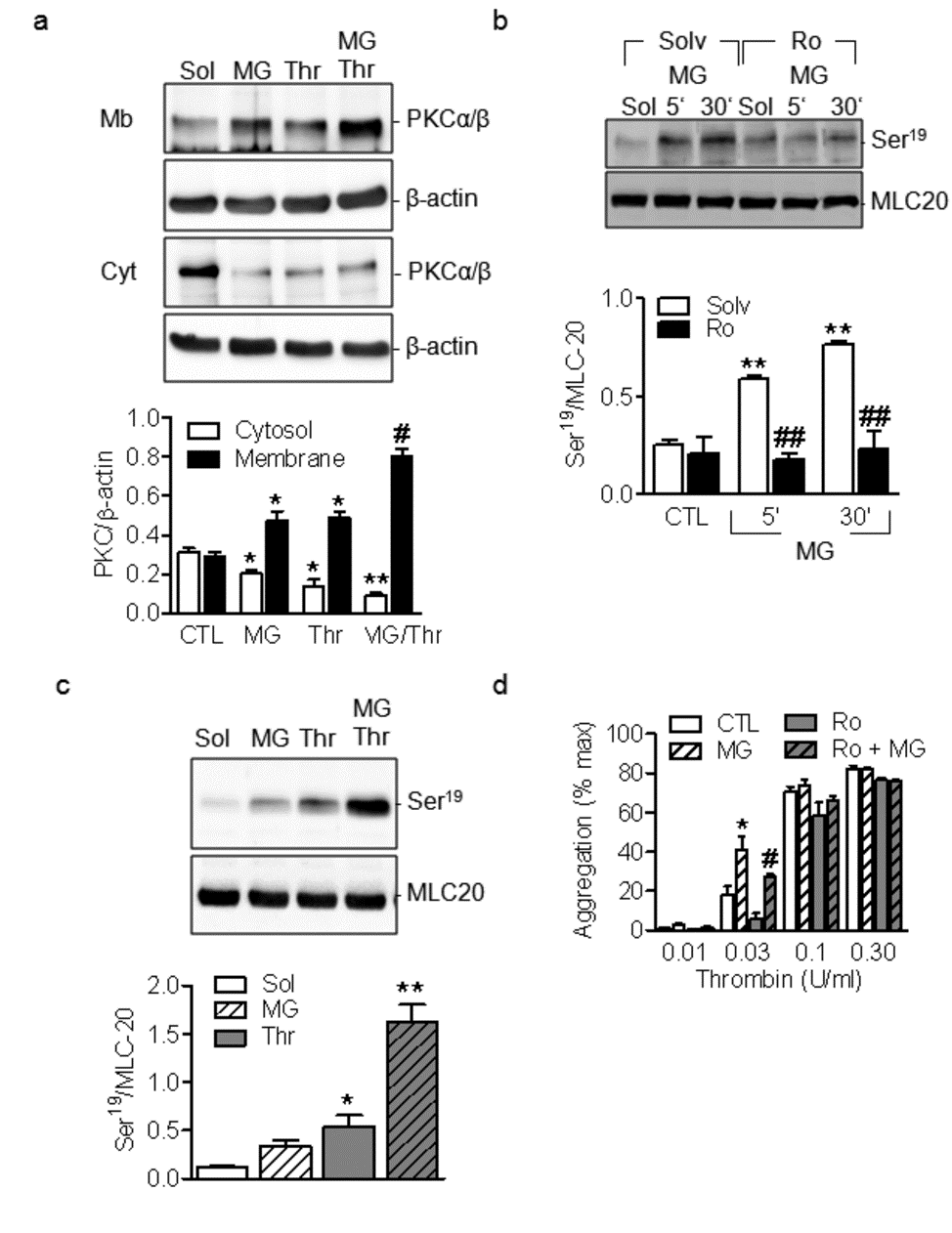
Effect of MG on PKC activation. (a) Membrane translocation of PKCα and β in washed human platelets stimulated with either methylglyoxal (MG, 1 mmol/L, 15 minutes) or thrombin (0.03U/ml) alone or in combination. (b) Effect of methylglyoxal (MG, 1 mmol/L, 15 and 30 minutes) on the phosphorylation of MLC20 in the absence or in the presence of the PKC inhibitor Ro-318820 (Ro, 300 nM, 30 minutes). (c) Effect of MG on thrombin-induced phosphorylation of MLC20. (d) Effect of Ro-318220 on the thrombin-induced aggregation of washed human platelets treated or not with MG. The graphs summarise the data from 6-8 different experiments; *P<0.05, **P<0.01 versus CTL and ^#^ P<0.05, ^# #^ P<0.01 versus agonists.

The activation of PKC was initially assessed by determining its translocation between the cytosol and membrane. Using specific antibodies we found that MG induced the membrane translocation of PKCα/β and potentiated the response to thrombin (Figure 3A). Protein kinase C α/β are of particular interest in platelets since they phosphorylate and inhibit the myosin light chain (MLC) phosphatase, and regulate the phosphorylation of MLC-20; which is important for the cytoskeletal reorganization that takes place during platelet aggregation and adhesion. We found that MG alone significantly enhanced the phosphorylation of MLC20 and that this effect was sensitive to PKC inhibition (Figure 3B). Moreover, MG potentiated the thrombin-induced increase in MLC20 phosphorylation (Figure 3C). Since Rho kinase can also regulate MLC20 phosphorylation we tested the effect of the Rho kinase inhibitor Y27632 on MG-induced MLC20 phosphorylation and found that Y27632 did not affect the response to MG while it inhibited the response to agonists such as thrombin and thromboxane A2 (Figure S2). The PKC inhibitor, R0-318220 (300 nmol/L) reversed the MG-induced increase in thrombin-induced platelet aggregation (Figure 3D).

### Effect of MG on platelet adhesion, spreading and thrombus stability

**Figure 4:**
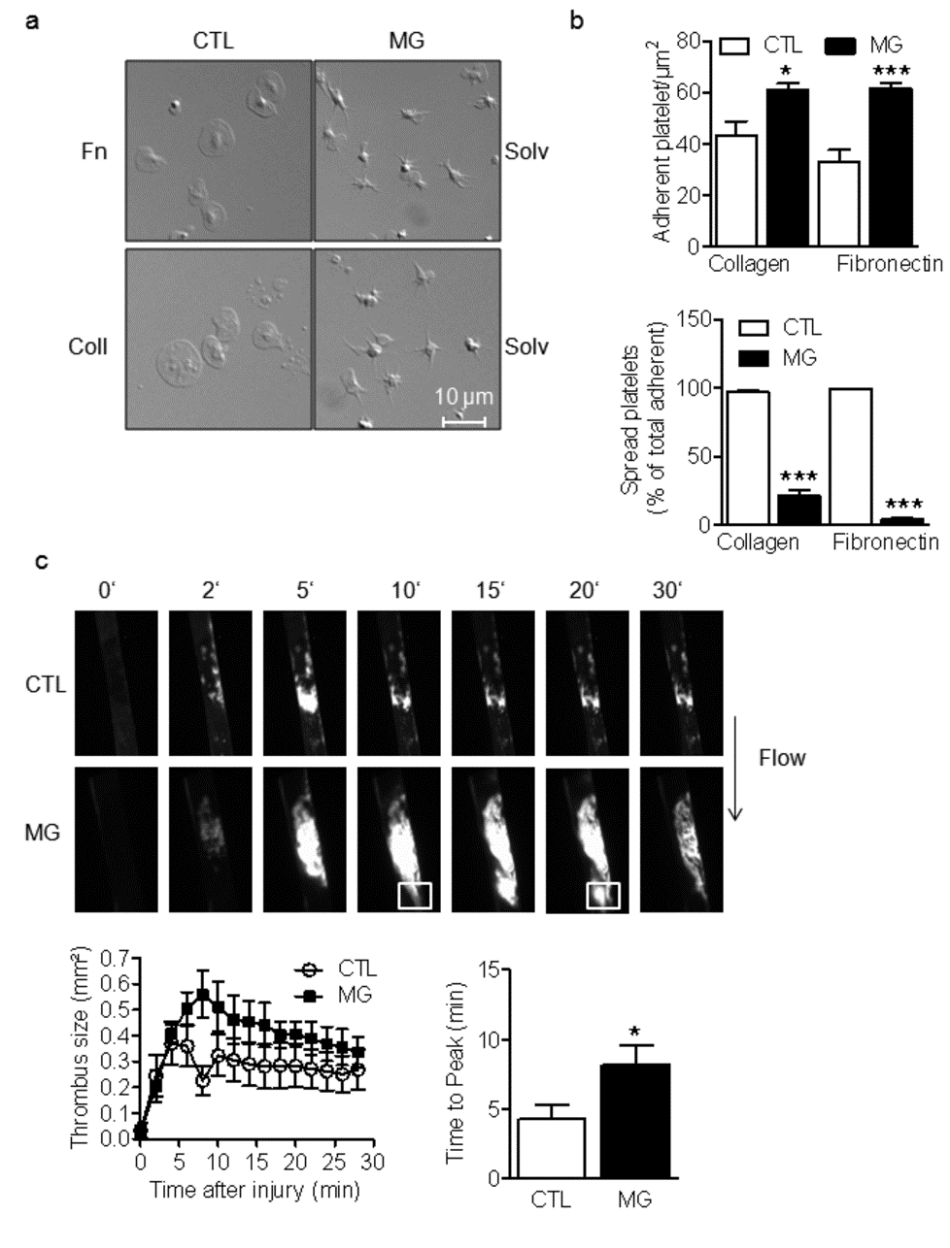
Effect of MG on platelet adhesion, spreading and *in vivo* thrombus formation. (a) Representative pictures and (b) quantification of adherent and spread washed human platelets (to fibronectin (Fn)- or collagen (coll)-coated slides) pre-treated with either solvent (CTL) or methylglyoxal (MG, 1 mmol/L, 15 minutes). (c) Representative pictures (upper panel) and quantification (lower graphs) of the effect of *in* *vivo* treatment of healthy mice with MG (1 mmol/L, 15 minutes) on thrombus size and time to peak after FeCl_3_-induced injury of carotid artery. The graphs summarize data obtained in platelets from 12 subjects or 6 animals per group; *P<0.05, ***P<0.001, versus CTL.

When exposed to the extracellular matrix, platelets adhere, develop filopodia and then spread [15]. MG significantly enhanced the number of platelets that adhered to both collagen and fibronectin but subsequently inhibited platelet spreading even after 60 minutes of incubation (Figure 4A).

Since platelet adhesion and spreading at sites of vascular injury is essential for hemostasis and thrombus stabilization, we next investigated the effect of MG on thrombus formation *in vivo* after FeCl_3_-induced injury of the carotid artery. Formation of the initial thrombus was not significantly different in vehicle and MG-treated mice but the thrombus was larger in the MG-treated group (Figure 4B) and was also less stable. Indeed, emboli frequently detached from the primary thrombus (see squares in Figure 4B). MG-treated mice also have a significantly shorter bleeding time (46.4±11.55s) compared to untreated control mice (151.8±30.26s). Moreover, re-bleeding was observed in 50% of the MG-treated mice confirming the formation of unstable clot.

### MG inhibits the phosphorylation of β3-integrin

**Figure 5:**
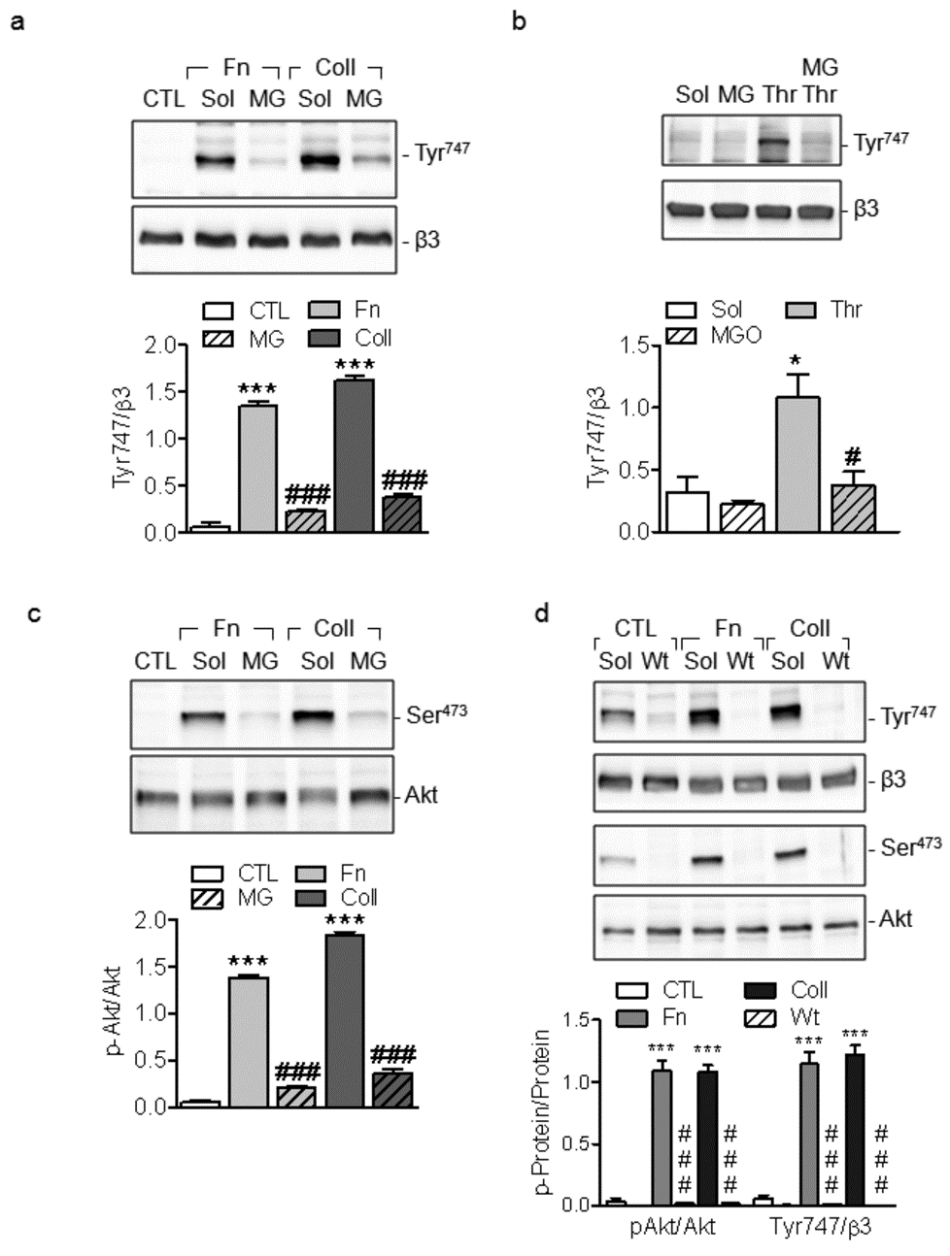
Effect of MG on the phosphorylation of β3 integrin and Akt. (a) Effect of MG (MG, 1 mmol/L, 15 minutes) on fibronectin (Fn) and collagen (coll)-induced tyrosine phosphorylation of β3 integrin (Tyr747). (b) Effect of MG on thrombin -induced tyrosine phosphorylation (Tyr747) of β3 integrin in washed human platelets. (c) Effect of MG on fibronectin (Fn) and collagen (coll)-induced phosphorylation of Akt (Ser 473). (d) Effect of wortmannin (Wt, 20 nmol/L, 30 minutes) on fibronectin (Fn) and collagen (coll)-induced phosphorylation of β3 integrin (Tyr747) and Akt (Ser 473). The graphs summarise the data from 6 different experiments; *P<0.05, ***P<0.001 versus sol or CTL and ^#^ P<0.05, ^# # #^ P<0.001 versus agonists.

Platelet adhesion leads to the tyrosine phosphorylation of αIIbβ3 integrin which transmits the outside in signal that is important for platelet spreading and thrombus stabilization. Given that the latter were impaired by MG, we assessed the effects of the compound on β3 integrin phosphorylation. The adhesion of human platelets to fibronectin or collagen significantly enhanced β3 integrin tyrosine phosphorylation (Figure 5A), an effect that was not observed in platelets pre-treated with MG. These effects were not dependent on adhesion *per se* as MG also attenuated the tyrosine phosphorylation of β3 integrin in platelet suspensions treated with thrombin (Figure 5B). MG did not affect either the basal or the agonist-induced increase in the expression of active β3 integrin on platelet surface, suggesting that MG did not act upstream of the integrin inside-out signaling (Figure S3).

The phosphatidylinositol 3-kinase (PI3K) plays an important role in regulating the function of integrin αIIbβ3 [16], therefore, we assessed the effects of MG on the agonist-induced phosphorylation of the PI3K downstream target Akt. While Akt phosphorylation (on Ser473) was enhanced in platelets adherent on collagen and fibronectin, pre-incubation with MG attenuated Akt phosphorylation (Figure 5C). Given that PI3K can act upstream as well as downstream of the integrin αIIbβ3 outside-in signaling we tested the effects of the PI3K inhibitor wortmannin on β3 integrin phosphorylation. Wortmannin significantly inhibited fibronectin and collagen-induced phosphorylation of Akt and β3 integrin (Figure 5D) suggesting that the β3 integrin activation is downstream of PI3K.

## Discussion

The results of the present investigation indicate that MG exerts a dual effect on platelet activation: a pro-aggregatory and pro-thrombotic effect linked to an increase in [Ca^2+^]_i_, and activation of classical PKC pathway, and an anti-spreading effect that involves the inhibition of PI3K/Akt pathway and the β3 integrin outside-in signaling.

While the deleterious effects of MG have been mostly linked to its rather slow formation and activation of RAGE [17] the results of the present study indicate that MG can acutely alter platelet reactivity and function. Such an acute effect can be a direct consequence of a rapid increase in blood MG concentration, such as that after a meal or during the so-called hyperglycemic spikes. Certainly, the hyperglycemic spikes that are often observed in patients with poorly managed diabetes have been shown to enhance the risk of developing cardiovascular complications. Moreover, acute hyperglycemia has been reported to enhance shear stress-induced platelet activation in patients with type II diabetes [18]. Depending on the method used to determine MG levels, its concentration in plasma has been reported to be significantly higher (up to 6 times) in patients with type 2 diabetes compared to healthy donors. For example, 0.4 mM MG has been measured in diabetic plasma using GC-MS and mM levels can be reached postprandially [5]. Our failure to measure MG in plasma using a commercially available ELISA kit suggests that either this assay method is inappropriate for the detection of MG in plasma or that MG could be only detected in fresh samples. Given that the intracellular concentration of MG is thought to be higher than the circulating levels it is also very likely that platelets from healthy vs diabetic patients mainly differ on their intracellular MG levels. In our study, the use of 1 mM concentration of MG for short-time stimulation simulates the postprandial burst of MG encountered in diabetic patients. This concentration is close to the pathophysiologically relevant range and has frequently been used by others investigating the biological actions of MG [19,20]. Our finding that MG was unable to affect the aggregation of PRP from diabetic patients may be explained by the fact that diabetic platelets which are permanently exposed to high plasma and/or intracellular concentration of MG *in vivo* are hyperreactive and do not further react to the addition of MG *in vitro*.

Regarding its mechanisms of action, we found that MG on its own was able to enhance platelet [Ca^2+^]_i_, activate PKC and enhance the phosphorylation of MLC20. The first steps in this process e.g. binding to an extracellular or intracellular mediator are currently unknown. However, since the acute application of G-HSA failed to reproduce the effects of MG the involvement of RAGE seems unlikely. Another possibility is that MG may bind to a non-RAGE receptor to mediate its effects as it has been characterized as a GABA_A_ receptor agonist [21] and can directly affect the function of the voltage-gated sodium channel Na _v_1.8 [7]. An extracellular receptor may, however, not be required as MG is membrane permeable [19,22]. Finally, the fact that the potentiating effect of MG was only evident at low concentrations of agonists and was surmounted by high concentrations of agonists points towards a competitive interaction of MG with a common intracellular signaling pathway.

The effect of MG on platelet [Ca^2+^]_i_ was paralleled by the activation of classical PKCs, and the phosphorylation of MLC20. Phosphorylation of MLC20 is thought to be one of the primary steps in the activation of actomyosin contractile events, important for platelet shape change and granule secretion [23,24]. Although calcium- and calmodulin-dependent myosin light chain kinase (MLCK) has been initially considered to be the primary regulator of MLC20 phosphorylation, it is well accepted to be regulated by the inhibition of the MLC20 phosphatase. The latter involves both the Rho kinase and PKCs. We were able to demonstrate that contrary to agonist-induced MLC20 phosphorylation, which was predominantly Rho-kinase dependent, the MG-induced effect primarly involves PKC.

The release of granule contents is an important step in the transmission and perpetuation of signaling between platelets. Given that it is tightly regulated by PKC [13] it was logic to look at the effect of MG on platelet degranulation. Interestingly, despite its ability to activate PKC, MG did not directly affect the degranulation of either dense or α-granules, but enhanced the thrombin-induced secretion of dense granule contents. Exactly why a substance that clearly affects PKC activity had no effect on degranulation is unclear but the level of PKC activation may have been below the threshold required to promote platelet degranulation.

Perhaps the most impressive effect of MG was its ability to prevent the spreading of platelets on collagen and fibronectin. Platelet activation with agonists such as thrombin or via the binding of β1 integrin to matrices during platelet adhesion leads to the initiation of common signaling events that finally result in a conformational change and αIIbβ3 integrin activation (inside-out signaling). The active integrin can then bind fibrinogen and transmit an outside-in signaling resulting in platelet spreading and thrombus stabilization [25]. How could MG interfere with the mechanisms involved in integrin outside-in signaling? It clearly prevented the tyrosine phosphorylation of β3 integrin and this effect could be attributed to the activation of the PI3K/Akt cascade which is essential for platelet spreading [26,27,28,29] as well as for the phosphorylation and activation of β3 integrin [30]. That MG inhibits the activity of the PI3K was initially reported in pancreatic beta cells [22] where it has been suggested to be, at least in part, linked to GSK-3 activation. Whether the effect of MG in platelets also involve GSK-3 activation is however unclear. PI3K is a key enzyme in platelet activation which can act upstream and downstream of the β3 integrin. Our finding that despite the inhibition of PI3K, MG failed to affect the thrombin-induced expression of active β3 on the platelet surface (inside-out signaling) suggests that the MG-mediated PI3K inhibition is likely to be downstream of integrin inside-out signaling. Given that the activation of PI3K by β3 integrin outside-in signaling has been reported to be important for thrombus stabilization [31,32], it was expected that MG which interferes with PI3K and platelet spreading would have significant consequences for thrombus formation and stability. Indeed, it was exactly what was observed *in vivo* on MG-treated mice which developed large but unstable thrombin. A phenomenon similar to what we previously reported in diabetic mice [33]. However, given that diabetes is associated with increased circulating levels of insulin which is known to be a potent activator of the PI3K/Akt pathway, the diabetes-associated changes in platelet function are likely the result of a complex interplay between insulin-induced hyperactivation and MG-induced inhibition of the Akt pathway.

Taken together, the present study shows that MG, by directly increasing platelet aggregability and decreasing thrombus stability, induces a pro-thrombotic phenotype in mice. Furthermore, the finding of the present study provides further evidence for the importance of MG in the pathogenesis of thrombotic complications encountered in diabetes. Given that an increased circulating MG has been also reported in different pathological conditions [34], understanding the molecular mechanisms of action of MG may help developing a targeted therapy to fight against its deleterious effect.

## Supporting Information

Figure S1 **Effect of mannitol on the thrombin-induced aggregation.** Washed human platelets were treated with either solvent (CTL) or mannitol (1 mmol/L, 15 minutes) prior to the stimulation with thrombin. The graphs summarise the data from at least 5 different individuals.
(TIF)

Figure S2 **Effect of Ro-318820 (Ro, 300 nM, 30 minutes) and Y27632 (Y27, 10 µM, 30 minutes) on the MG, thrombin and U46-induced phosphorylation of MLC20 in washed human platelets.** Identical results were obtained in 4 additional experiments.
(TIF)

Figure S3 **Effect of MG on the surface expression of the active** **β3 integrin**. Washed human platelets were incubated with either solvent or MG (1 mmol/L, 15 minutes) prior to the stimulation with either the thrombin receptor activating peptide (TRAP) or the thromboxane A2 analogue (U46619) and the surface expression of active β3 integrin was detected by flow cytometry. The graphs summarise the data from at least 5 different individuals. *P<0.05 versus CTL.
(TIF)
