# Supplementary figures and images for: Methylglyoxal Induces Platelet Hyperaggregation and Reduces Thrombus Stability by Activating PKC and Inhibiting PI3K/Akt Pathway
Source: PLoS One. 2013 Sep 13;8(9):e74401. doi: 10.1371/journal.pone.0074401 (PMC3772821; doi:10.1371/journal.pone.0074401)

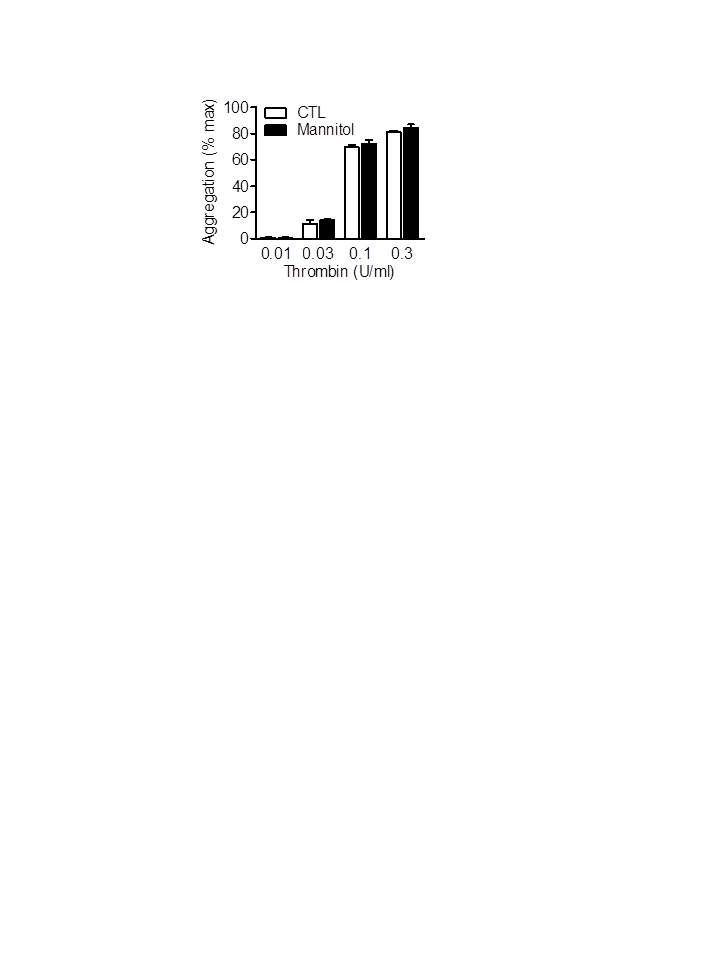

Supplement: Figure S1 — Effect of mannitol on the thrombin-induced aggregation. Washed human platelets were treated with either solvent (CTL) or mannitol (1 mmol/L, 15 minutes) prior to the stimulation with thrombin. The graphs summarise the data from at least 5 different individuals. (TIF) [file pone.0074401.s001.tif]

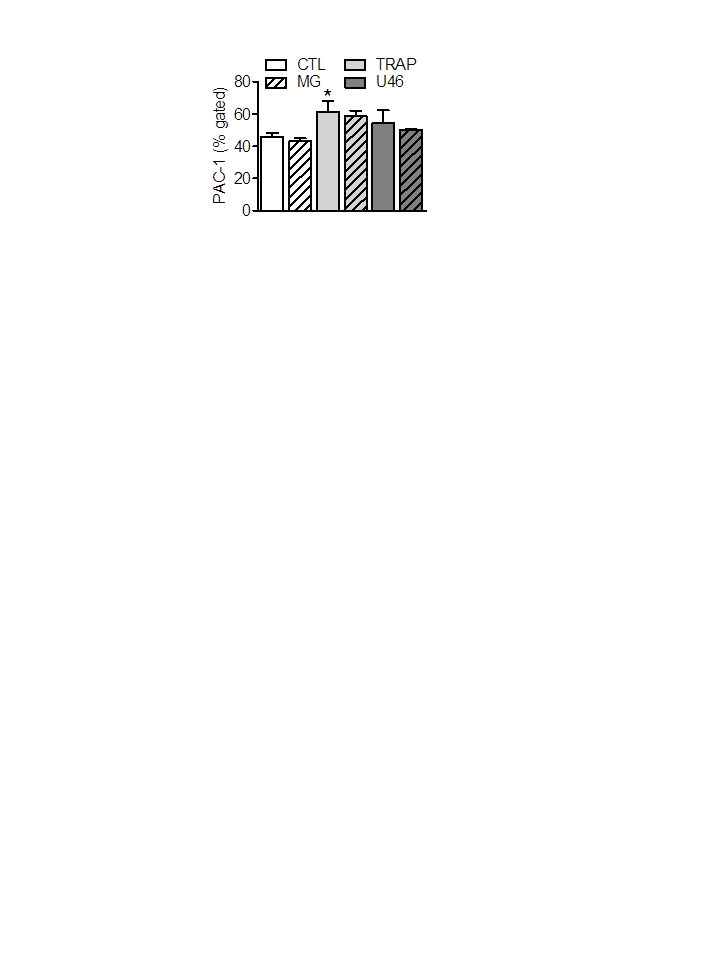

Supplement: Figure S3 — Effect of MG on the surface expression of the active β3 integrin. Washed human platelets were incubated with either solvent or MG (1 mmol/L, 15 minutes) prior to the stimulation with either the thrombin receptor activating peptide (TRAP) or the thromboxane A2 analogue (U46619) and the surface expression of active β3 integrin was detected by flow cytometry. The graphs summarise the data from at least 5 different individuals. *P<0.05 versus CTL. (TIF) [file pone.0074401.s003.tif]
